# The Prognostic Relevance of the Proliferation Markers Ki-67 and Plk1 in Early-Stage Ovarian Cancer Patients With Serous, Low-Grade Carcinoma Based on mRNA and Protein Expression

**DOI:** 10.3389/fonc.2020.558932

**Published:** 2020-10-07

**Authors:** Franz Rödel, Shengtao Zhou, Balász Győrffy, Monika Raab, Mourad Sanhaji, Ranadip Mandal, Daniel Martin, Sven Becker, Klaus Strebhardt

**Affiliations:** ^1^Department of Radiotherapy and Oncology, University Hospital, Goethe-University, Frankfurt am Main, Germany; ^2^Frankfurt Cancer Institute, Goethe-University, Frankfurt am Main, Germany; ^3^German Cancer Research Center (DKFZ), Heidelberg, Germany; ^4^German Cancer Consortium (DKTK) Partner Site: Frankfurt, Frankfurt am Main, Germany; ^5^State Key Laboratory of Biotherapy, Department of Obstetrics and Gynecology, West China Second Hospital, Sichuan University, Chengdu, China; ^6^Department of Bioinformatics and 2nd Department of Pediatrics, Semmelweis University, Budapest, Hungary; ^7^TTK Cancer Biomarker Research Group, Budapest, Hungary; ^8^Department of Gynecology, University Hospital, Goethe-University, Frankfurt am Main, Germany

**Keywords:** polo-like kinase 1, Ki-67/MIB1, ovarian cancer, prognosis, mRNA and protein expression

## Abstract

Since type and duration of an appropriate adjuvant chemotherapy in early-stage ovarian cancer (OC) are still being debated, novel markers for a better stratification of these patients are of utmost importance for the design of an improved chemotherapeutical strategy. In contrast to numerous cancer studies on cellular proliferation based on the immunohistochemistry-driven evaluation of protein expression, we compared mRNA and protein expression of two independent markers of cellular proliferation, Ki-67 and Plk1, in a large cohort of 243 early-stage OC and their relationship with clinicopathological features and survival. Based on marker expression we demonstrate that early-stage OC patients (stages I/II, low-grade, serous) with high expression (Ki-67, Plk1) had a significantly shorter progression-free survival (PFS) and overall survival (OS) compared to patients with low expression (Ki-67, Plk1). Remarkably, based on mRNA expression this significant difference got lost in advanced stages (III/IV): At least for PFS, high levels of Ki-67 and Plk1 correlate with moderately better survival compared to patients with low expressing tumors. Our data suggest that in addition to Ki-67, Plk1 is a novel marker for the stratification of early-stage OC patients to maximize therapeutic efforts. Both, Ki-67 and Plk1, seem to be better suited in early-stages (I/II) as therapeutical targets compared to advanced-stages (III/IV) OC.

## Introduction

OC represents the most common lethal gynecological malignancy (1) and continues to cover a clinical challenge despite advances in the mechanistical knowledge of tumorigenesis and disease progression (2). Early-stage OC (stages I/IIa) contributes with 25–20% to the total number of cases (3). In these cases, the staging is based on a retroperitoneal inspection including lymph node dissection according to the National Comprehensive Cancer Network (NCCN) guidelines. While most women with low-grade tumors (grade 1) don't need treatment after surgery, patients with grade 2 tumors are closely watched following surgery or are treated with chemotherapy including carboplatin and paclitaxel for 3–6 cycles. As alternatives, carboplatin can be replaced by cisplatin, and docetaxel can be replaced by paclitaxel. For stage II cancers, chemotherapy for at least six cycles follows surgery. A recent study including 1,110 patients with early-stage OC revealed that OS with stage II disease was poorer than that of patients with stages Ia/b or Ic disease (4). Despite a good likelihood of a complete surgical removal of tumor tissues by removing the internal genitals, the 10-year PFS ranges between 56 and 78% (5). This relatively low figure indicates that at the time of primary surgery a sub-clinical spread of tumor has already occurred. A rigorous search for occult metastases induced by peritoneal seeding of cancer cells is of utmost importance for an accurate surgical staging and for the choice of a tailored therapeutical strategy of OC patients in early stages. However, in patients with similar tumor stage and surgical or cytotoxic treatment, variations in outcome can be observed.

Different investigations explored the role of age, ascites, stage, grade and tumor type in early-stage OC. A study by Young et al. (6) revealed that patients diagnosed in stages Ia/b with grade 1 (well-differentiated) tumors or grade 2 (moderately differentiated) tumors had a similar 5-year OS (94 vs. 98%). After a median follow-up of at least 6 years a significant difference in 5-year progression-free survival (PFS) between patients given no therapy and those treated with melphalan could not be observed. The authors concluded that a comprehensive staging at the time of surgical resection is required to identify those patients who can be followed without adjuvant chemotherapy. Moreover, the multivariate analysis of a large cohort of 1,545 patients indicated that grade is a powerful prognostic indicator of PFS comparing grade 2 vs. grade 1 disease (7). Furthermore, in a study of the British Columbia Cancer Agency including 605 cases of early-stage OC the independent prognostic significance of age, ascites, stage and tumor type was confirmed (8). Interestingly, Hsieh et al. (9) analyzed very recently in a retrospective investigation a cohort of 437 patients suffering from early-stage OC and uncovered that the FIGO (International Federation of Gynecology and Obstetrics) stage, histologic type and tumor grade are significant prognostic factors of 5-year disease-free survival (DFS). Using a multivariate Cox regression model, it could be shown that only the FIGO stage represents a significant prognostic factor for 5-year OS. The analysis indicated better DFS and OS for low-grade (grade 1/2) patients compared to high-grade tumors (grade 3). This study supported a survival benefit of a taxane-based adjuvant chemotherapy compared to non-taxane-based chemotherapy like platinum cyclophosphamide. Despite these insights, the discussion about reliable prognostic markers is still ongoing.

Two randomized trials, “Action” (EORTC) and “ICON 1” (International Collaborative Ovarian Neoplasm 1) were performed to address the issue of a debatted survival benefit of immediate adjuvant chemotherapy in early disease. These studies did not provide a clear guideline for the systemic treatment of patients with early-stage disease (10, 11). While the long-term follow-up of patients participating in the ICON 1 study indicated the efficacy of adjuvant chemotherapy for early-stage OC, the benefit was more pronounced in the high-risk group with stage Ib/c (grades 2/3), and stage I (grade 3) and FIGO stage II (12). Several follow-up studies supported the conclusion that adjuvant chemotherapy did not improve survival for early-stage OC patients with optimal surgical staging (13–15). Despite a favorable prognosis for early-stage OC patients a considerable heterogeneity in regard to the risk of relapse varying between 15 and 40% was determined in different clinical trials (7, 16–18). Multiple risk factors including age, FIGO stage, grade, histological subtype and ascites have already been identified. Prognosis is better in early disease, but worse in the advanced stage, compared to high grade carcinomas which is mainly due to inadequate response to platinum-based chemotherapy Although a surgical staging plays a key role for an optimal design of adjuvant chemotherapy, a comprehensive staging procedure is complicated and a challenging task. Therefore, the identification of novel markers and risk factors for relapse that support the surgical staging is of general importance to design tailored strategies for an improved prognosis of early-stage OC patients.

In this study we compared the prognostic potential of two markers of cellular proliferation in early-stage OC, Ki-67 and Polo-like kinase 1 (Plk1) at the levels of mRNA and protein expression. Of several proliferation markers, the nuclear protein Ki-67, is the most frequently studied marker for cellular proliferation in human tissues (19). Ki-67 plays a key role to keep mitotic chromosomes dispersed in the cytoplasm following their dissociation from the nuclear envelope (20). Ki-67 might function as a surfactant at the phase boundary between cytoplasm and mitotic chromatin. The fact that the Ki-67 protein is expressed during all active cell-cycle stages (G1, S, G2, mitosis), except G0, makes it a versatile tool to determine the percentage of proliferating cells within tissues. Traditionally, cancer proliferation can be assessed using immunohistochemical staining of nuclear antigen Ki-67 (21–23). The prognostic role of Ki-67 was explored in a broad spectrum of cancer types, including brain, breast, neuroendocrine, and lymphoid cancers, where the Ki-67 stainingcontributed to the grading of tumors.

Polo-like kinase 1 (Plk1), belonging to the enzyme family of polo-like kinases (24–26), is a multifunctional regulator of cell-cycle progression (27–29), which has been widely shown to be overexpressed in a multitude of cancer entities including OC (30, 31) and to correlate with poor patient prognosis (32–35). In addition, inhibition of Plk1 in multiple animal models and clinical trials revealed Plk1 as valuable target for cancer therapy (35–38), with a first clinical study to indicate comparable anti-tumor activities of the small molecule Plk1 inhibitor Volasertib (BI6727) and single agent paclitaxel in advanced OC (39). Despite an intensive use of Ki-67 and Plk1 for an immunohistochemical analysis of a huge variety of cancer entities, their prognostic potential in early-stage OC remains elusive.

## Materials and Methods

### Database Construction

To identify datasets suitable for the analysis we searched GEO (https://pubmed.ncbi.nlm.nih.gov/) and TCGA (http://cancergenome.nih.gov) repositories. In this search, the keywords “ovarian,” “cancer,” “treatment,” “response,” and “survival” were used. We only included publications with available raw microarray gene expression data, clinical treatment, response or survival information, and at least 20 patients enrolled. Only three closely related microarray platforms, GPL96 (AffymetrixHG-U133A), GPL570 (Affymetrix HG-U133 Plus 2.0), and GPL571/GPL3921 (Affymetrix HG-U133A 2.0), were considered.

### Pre-processing

First, the raw CEL files were MAS5 normalized in the R statistical environment (www.r-project.org) using the Affy Bioconductor library (21). This was followed by a second normalization to set the average expression of the 22,277 identical probe sets in each chip to 1,000 (22). Normalized gene expression and clinical data were integrated into a PostgreSQL relational database.

### Kaplan-Meier Plotter

The prognostic significance of Plk1 (Affymetrix ID: 202240_at) and Ki-67 (Affymetrix ID: MKI-67 212023_s_at) mRNA expression was evaluated by the online Kaplan-Meier Plotter (www.kmplot.com) (40). This platform covers gene expression data and survival information of OC patients from Gene Expression Omnibus database. Based on an auto-select, best cut-off option, patients were split into groups covering early-stage I/II (excluding G3 tumors) and advanced-stage III+IV tumors displaying a low and a high Plk1 and Ki-67 mRNA expression. Log rank *p*-values and hazard ratio (HR) with 95% confidence intervals were calculated and given in each plot. In total, a number of 58 stage I/stage II patients with PFS data and 61 patients with OS data available, and 984 stage III/stage IV patients with PFS data and 1,145 patients with OS data available were included in the analyses. All patients were treated with optimal debulking surgery and received platinum-based chemotherapy, and more than 50% of the patients were treated with taxane. Other groups of patients received gemcitabine, topotecan, paclitaxel, docetaxel, or avastin.

### Patient's Characteristics

All patients enrolled were from West China Second Hospital, Sichuan University. Following an institutional review board approval (Ethics Committee of Sichuan University, Ethics approval no. 20180928032) in accordance with the Helsinki Declaration of 1975 and after obtaining written informed consent, a total of 243 patients were included for immunohistochemical staining. Patients were routinely subjected to standard pre-treatment staging including computer tomography or magnetic resonance tomography of the pelvis and abdomen, chest radiography, and baseline laboratory studies. In all early-staged OC patients a complete peritoneal and retroperitoneal staging was performed at a single institution to exclude a bias of differing staging approaches. The median age was 51 years with a range of 34–85 years. Sixty five patients presented with stage I (26.7%) and 178 patients (73.3%) with stage II disease. All patients enrolled were of serous pathological subtype and displayed a low-grade differentiation. Median follow-up for all patients was 32 months (range: 12–109 months). Patients were treated between 2006 and 2015. All patients enrolled were R0 so that the survival data are comparable. We combined Ca-125 and CT scan results together for patient follow-up.

### Treatment and Follow-Up Assessment, Sensitivity to Chemotherapy

Treatment of the patients including paclitaxel/carboplatin-based chemotherapy and schedule of follow-up examination was performed rigorously based upon NCCN guidelines. Sensitivity to platinum chemotherapy was either defined as a significant decline of serum CA-125 level in the follow up examination, while resistance was defined as steady-state levels with no decline of the marker or was defined as steady-state levels with no decline of the marker or recurrence before 6 months to the end of platinum-based chemotherapy, or platinum-refractory (progression during initial therapy) disease.

### Histochemical Staining and Scoring for Plk1 and Ki-67 Expression

Production of tissue microarrays (TMA) was performed at West China Second Hospital, Sichuan University. The accuracy of the pathological diagnosis of all patients included was confirmed by at least two independent pathologists in a double-blinded manner. Next, TMA-slices were subjected to a standardized horseradish peroxidase/3,3-diaminobenzidine (DAB) chromogen technique with primary anti-Plk1 antibodies (Santa Cruz Biotechnology) at a 1:100 dilution and anti Ki-67/MIB1 antibodies (Agilent Dako) at a 1:50 dilution. Next, two investigators (FR, MJ) performed scoring without knowledge of the clinicopathological or clinical data. Plk1 immunoreactivity was assessed considering the fraction of Plk1-positive tumor cells [1: (0–25%), 2: (26–50%), 3: (51–75%) and 4: (>75%)] and the intensity of staining scored as 1+ (weak), 2+ (moderate) and 3+ (intense). Finally, these parameters were multiplied to gain an individual weighted score (WS). A WS ≤ 6 was defined as “low” and a WS of >6 as “high” Plk1 expression. The Ki-67 index was defined as percentage of Ki-67 nuclear positive cancer cells in 10 representative microscopic fields and is given as percentage of Ki-67 positive cells/total tumor cells.

### Statistical Analysis

Assessment of the correlation between marker expressions was performed using the Spearman's correlation coefficient. Progression-free survival (PFS) and overall survival was defined as the time from tumor surgical resection to any disease progression (local, regional, or distant), death by OC or any reasons or the day of the last follow-up. Survival was recorded according to the Kaplan-Meier method. Univariate and multivariate analyses were computed using the log-rank test and the Cox proportional hazard model, respectively. A *p* < 0.05 was considered statistically significant. IBM SPSS Version 25 (IBM, Ehingen, Germany) was used for all statistical analyses.

## Results

### Plk1 and Ki-67 Gene Expression and Survival of OC Patients

**Figure 1:**
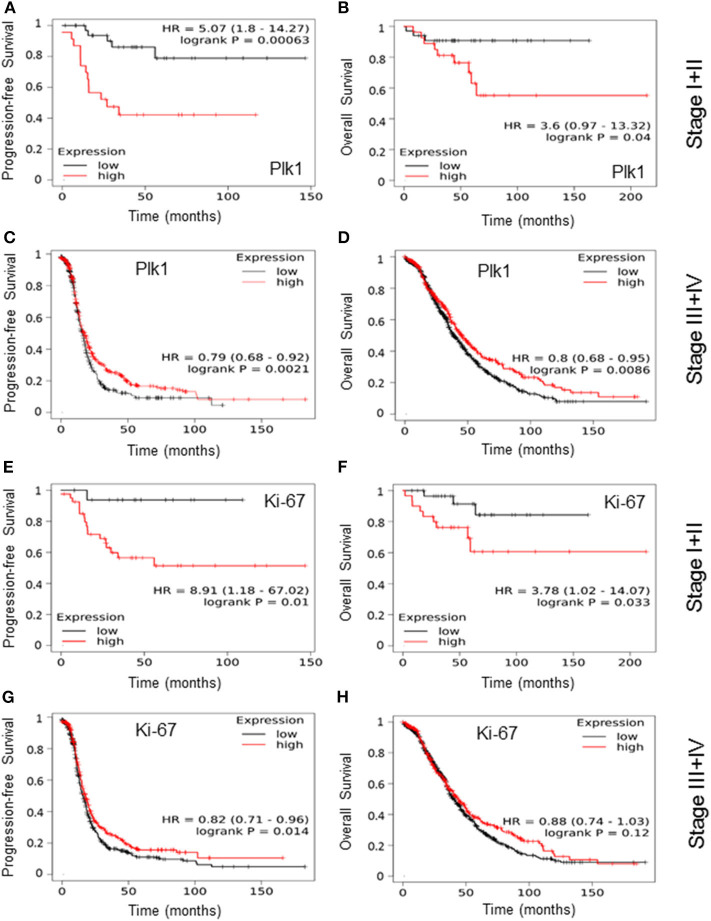
Based on the best cut-off option, patients were split into groups of a low and a high Plk1 and Ki-67 mRNA expression and survival curves for progression-free survival and overall survival were plotted for early-stage I+II **(A,B,E,F)** and advanced-stage III+V **(C,D,G,H)** tumors of patients with OC treated with debulking surgery and CT using the Kaplan Meier plotter database. Log rank *p*-values and hazard ratio (HR) with 95% confidence intervals were given in each plot.

First, to study the clinical impact of Plk1 and Ki-67 mRNA expression in OC patients, we evaluated the correlation between these markers and patients' survival based on Kaplan Meier plotter analyses according to our previous investigations (40). Survival curves were plotted for stage I+II and stage III+IV tumors to discriminate early and advanced stage disease. A high Plk1 mRNA expression was found to be correlated to a significantly impaired PFS [HR 5.07 (1.8–14.27), *P* = 0.00063, Figure 1A] and OS [HR 3.6 (0.97–13.32), *P* = 0.04, Figure 1B] in early-stage I+II malignancies. By contrast, for stage III+IV patients we observed an inverse characteristic with a high Plk1 expression to correlate with better PFS [HR 0.79 (0.68–0.92), *P* = 0.0021, Figure 1C] and OS [HR 0.80 (0.68–0.95), *P* = 0.0086, Figure 1D]. In this patient cohort, upper quartile survival was 16 months (low expression) vs. 17.5 months (high expression) for PFS and 38.73 (low expression) vs. 45.23 months (high expression) for OS. For patients with stage I+II tumors and a high Ki-67 mRNA expression we also observed a significantly impaired PFS [HR 8.91 (1.18–67.02), *P* = 0.01, Figure 1E] and OS [HR 3.78 (1.02–14.07), *P* = 0.033, Figure 1F]. Again, for stage III+IV patients an inverse characteristic with a high Ki-67 expression to correlate with better PFS [HR 0.82 (0.71–0.96), *P* = 0.014, Figure 1G] was evident, while for OS [HR 0.88 (0.74–1.03), *P* = 0.12, Figure 1H] no significant correlation was to be observed. In these patients, upper quartile survival was 15.87 months (low expression) vs. 17.5 months (high expression) for PFS and 40.4 (low expression) vs. 43.97 months (high expression) for OS.

### Immunohistochemical Analysis of Plk1 and Ki-67 Expression and Survival in Early-Stage OC Patients

**Figure 2:**
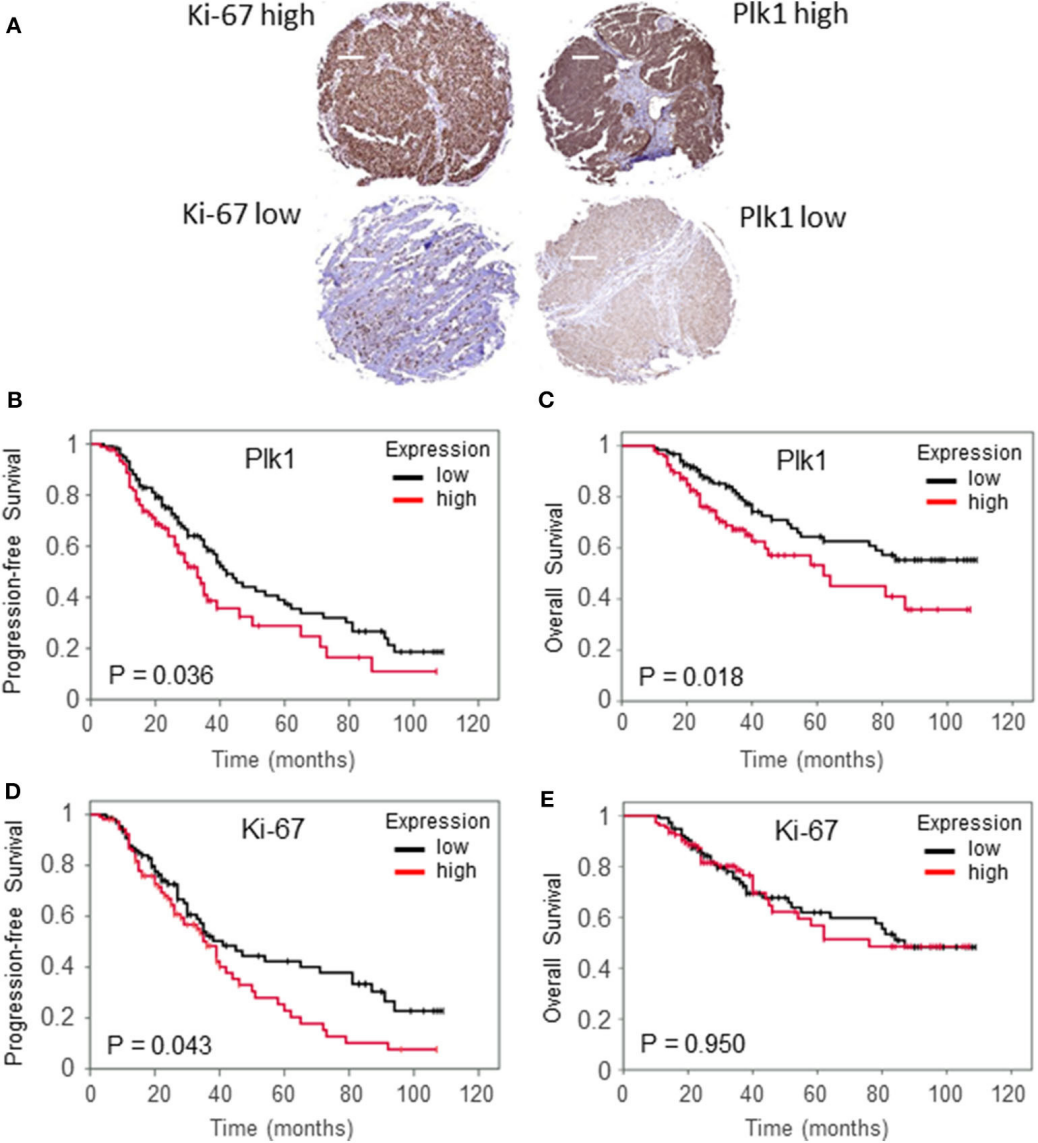
Examples of ovarial cancer biopsies with high and low immunohistochemical detection of Ki-67 and Plk1 **(A)**. Original magnification × 100, scale bar: 100 μm. Relapse-free survival **(B,D)** and overall survival **(C,E)** according to low Plk1 expression (WS ≤ 6) and low Ki-67 levels (≤20%) vs. high Plk1 expression (WS > 6) and high Ki-67 levels (>20%) in patients with early-stage ovarial cancer treated with debulk surgery and CT.

To confirm the findings on an inverse significant impact of both, Plk1 and Ki-67 high expression on clinical outcome in stage I+II tumors, we next performed an immunohistochemical staining of the markers in an independent cohort of patients. Characteristics of Plk1 expression and levels of Ki-67 detection based on the cut-off values cover 95 patients (43.61%) with a high (weighted score >6) and 123 (56.6%) with a low (weighted score: WS ≤ 6) Plk1 detection. Ninety five patients (48.07%) displayed high percentages of Ki-67 reactivity (cut-off value: 20%) and 116 patients (52.0%) low Ki-67 scores. While, examples of low and high Plk1 and Ki-67 immunohistochemical staining are given in Figure 2A, patient- and tumor-related characteristics according to Plk1 and Ki-67 detection are summarized in Table 1. For both biological markers, we did not observe a significant correlation with histopathological and clinical characteristics age, stage, and response to CT, nor did we recognize a correlation between Plk1 and Ki-67 expression. As depicted in Figures 2B,D, high levels of Plk1 [HR 1.48 (1.02–2.16), *P* = 0.036] and Ki-67 [HR 1.45 (1.00–2.11), *P* = 0.043] immunoreactivity were significantly related to PFS. A further marker with a significant impact on PFS in univariate analyses covers response to CT [HR 13, 6 (8.70–21.28), *P* = 0.0001], while only response to CT (*P* = 0.001) and Ki-67 expression (*P* = 0.050) remained significant for this endpoint in multivariate analyses (Table 2). Median PFS survival in these patients was 41.0 months (low expression) vs. 33.0 months (high expression) for Plk1 and 38.0 (low expression) vs. 35 months (high expression) for Ki-67.

**Table 1:** Clinicopathological findings according to Ki-67 and Plk1 expression.

	**No. of patients**	**Ki-67 ≤ 20% *n* (%)**	**Ki-67 > 20% *n* (%)**	***P*-value**	**No of patients**	**Plk1 WS ≤ 6 *n* (%)**	**Plk1 WS > 6 *n* (%)**	***P*-value**
**AGE**								
≤51 years >51 years	114 109	56 (25.1) 60 (26.9)	58 (26.0) 49 (19.9)	0.422	112 106	64 (29.3) 59 (27.0)	48 (20.0) 47 (21.6)	0.892
**STAGE**								
I II	57 166	31 (13.9) 85 (38.1)	26 (11.6) 81 (36.4)	0.756	57 161	35 (16.0) 88 (40.36)	22 (10.09) 73 (33.5)	0.438
**RESPONSE TO CT**								
Yes No	169 48	86 (39.6) 24 (19.9)	82 (64.5) 24 (18.9)	1.000	163 48	96 (45.4) 24 (11.8)	67 (31.7) 24 (11.8)	0.321
**KI-67**								
Low (≤20%) High (>20%)					113 103	65 (30.9) 58 (26.8)	48 (22.2) 45 (20.8)	0.891
**PLK1**								
Low (WS ≤ 6) High (WS > 6)	123 93	65 (30.0) 48 (22.2)	58 (26.8) 45 (20.8)	0.891				

*Clinicopathological findings according to PlK3 expression and pT273 caspase-8 levels*.

**Table 2:** Univariate and multivariate analyses of prognostic factors in patients with Ovarial Cancer.

			**Multivariate analyses**		
			**95% Confidence interval (CI)**		
	**Univariate *P*-value**	**Hazard ratio (HR)**	**Lower**	**Upper**	***P*-value**
**PROGRESSION-FREE SURVIVAL**					
Response to CT (yes/no)	**0.0001**	12.30	7.70	19.65	**0.0001**
Ki-67 (≤20%/>20%)	**0.043**	1.45	1.00	2.04	**0.050**
Plk1 (WS ≤ 6/>6)	**0.036**	1.39	0.95	1.85	0.084
**OVERALL SURVIVAL**					
Age (≤57/>57)	**0.048**	0.89	0.55	1.17	0.266
Response to CT (yes/no)	**0.0001**	7.83	4.96	12.36	**0.001**
Plk1 (WS ≤ 6/>6)	**0.018**	1.49	1.01	2.225	**0.045**

*Bold values indicate significant values*.

According to the Kaplan Meier analysis, patients with a high Plk1 score (WS > 6) revealed a significantly impaired OS [HR 1.74 (1.09–2.7), *P* = 0.018] compared to patients with a low Plk1 expression (Figure 2C). By contrast, Ki-67 detection does not correlate with OS [HR 1.01 (0.63–1.62), *P* = 0.950, Figure 2E]. Additional clinicopathologic factors with a significant impact on OS in univariate analysis included age [HR 1.62 (1.05–2.50), *P* = 0.048] and response to CT [HR 8.58 (5.07–14.53), *P* = 0.0001]. In multivariate analyses, response to CT (*P* = 0.001) and Plk1 expression (*P* = 0.045) remained significant independent factors, while age lost its significance for this endpoint (Table 2). Median OS survival encounter for 84.0 months (low expression) vs. 59.0 months (high expression) for Plk1 and 85.0 months (low expression) vs. 62.0 months (high expression) for Ki-67.

**Figure 3:**
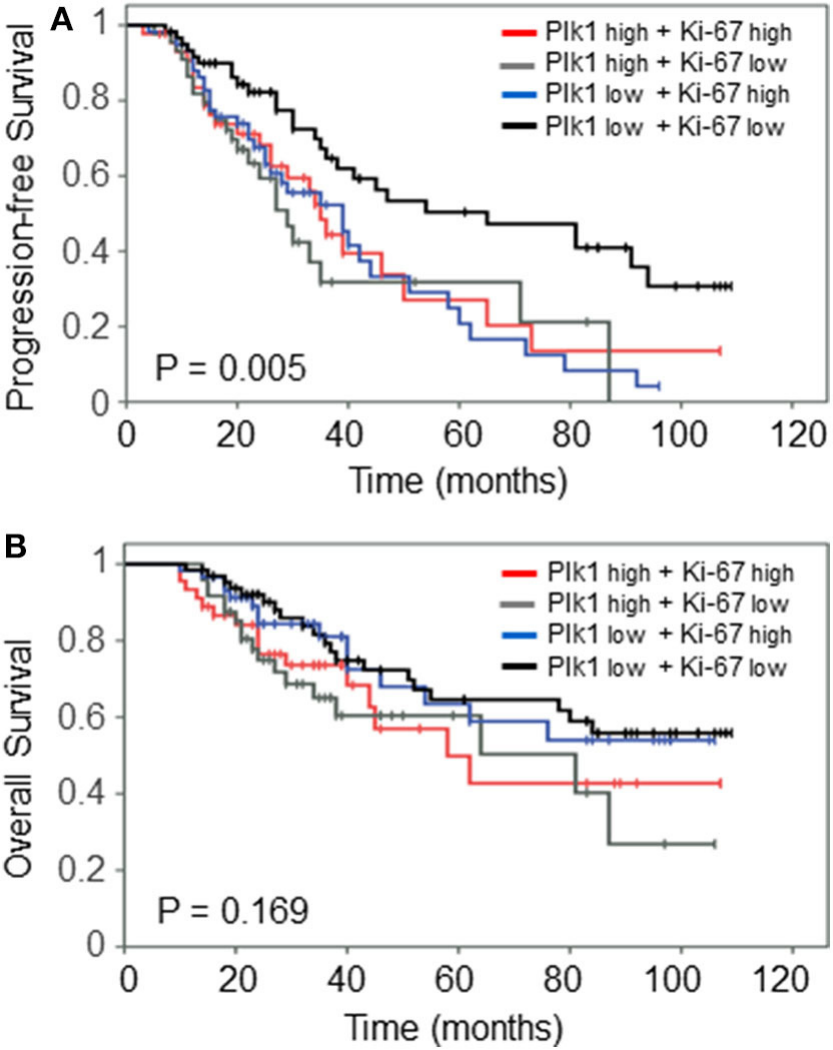
Prognostic impact of combined Plk1^high^/Ki-67^high^ vs. Plk1^high^/Ki-67^low^ vs. Plk1^low^/Ki-67^high^ vs. Plk1^low^/Ki-67^low^ expression on **(A)** relapse-free survival and **(B)** overall survival in the patients with early-stage I+II OC treated with debulking surgery and CT.

We finally asked whether a combined variable may be superior in predicting clinical outcome PFS and OS. Thus, we performed analyses on a combined Plk1 and Ki-67 variable. As illustrated in Figure 3A, patients with a Plk1^high^ and Ki-67^high^ or Ki-67^low^ expression presented a significantly (*P* = 0.005) impaired PFS as compared to patients with a Plk1^low^ and Ki-67^low^ detection. By contrast, combined Plk1/Ki-67 variables did not discrimate patients with a favorable/worse OS (*P* = 0.169, Figure 3B).

## Discussion

Increasing evidence indicates that OC is characterized by multiple chromosomal aberrations, mutations and molecular alterations (41) suggesting that a single therapeutical strategy will hardly be sufficient for all OC patients challenging the “one-size-fits-all” therapeutical approach. In regard to the toxicity, the expense, the inconvenience and the risk of second cancer, the definition of a group of patients with low aggressive tumors who do not need adjuvant therapy represents a substantial benefit. Multiple studies suggested a widely adopted treatment recommendation that patients diagnosed with OC stage Ia and grade 1 should not receive adjuvant therapy (42); many cancer centers include stage Ib, grade 1 in this low-risk group. However, grade assignments lack reproducibility and do not reflect the biological behavior of the different types. The group so defined at low risk that can avoid adjuvant therapy is rather small (5–10% of stage I and II ovarian carcinomas) indicating the need of robust tumor markers.

The markers Ki-67 and Plk1 both play an important role in tumor development, progression and in overall prognosis. Our previous investigations demonstrated that Ki-67 and Plk1 are expressed in a vast majority of tumor tissues examined (34, 43, 44). The findings led us to believe that both might be used as biomarkers for aggressive disease behavior and predictors of poor overall survival. The proliferation marker Ki-67 reflects the tumor cell proliferation rate as it correlates with progression, metastasis and prognosis in a number of different malignancies. In OC, several studies have indicated poor prognosis, when Ki-67 staining is strong. Still, the literature provides conflicting results about the prognostic potential of Ki-67 immunolabeling in OC (45–50), essentially because cut-off points are not consensual and the studies often include a broad range of clinical stages. However, the majority of these studies performed between 1998 and 2008 are in line with our results by demonstrating prognostic potential of Ki-67 in OC. More recent investigations also support the prognostic role of Ki-67. Chen et al. (51) described in 2016 a study on a cohort of 318 high-grade serous ovarian cancer (HGSOC) patients with a majority in advanced stage disease (FIGO stages III-IV, 77.1%). With a cut-off of 40%, low Ki-67 was significantly associated with decreased PFS (20 vs. 35%, 5-year PFS, *P* < 0.001) and decreased OS (31 vs. 55%, 5-year PFS, *P* < 0.001). Decreased PFS (HR 2.98, 95% CI 1.75–6.56, *P* < 0.001) and decreased OS (HR 1.75, 95% CI 1.38–5.01, *P* < 0.003) were confirmed by multivariate analysis. In our samples, we considered more than 20% stained nuclei to be positive, which is in the range of cut-offs from recent OC studies. Furthermore, in 2018 Sehouli et al. (52) published a cohort of 68 patients suffering from primary low-grade serous OC (LGSOC) analyzed for immunohistochemical Ki-67 staining by using an online tool cut-off finder. In the group of Ki-67 > 6.28% the median OS was found to be 66 months as opposed to 83 months for low Ki-67 staining (Ki-67 < 6.28%). Using an optimal cut-off of 1.85% for the analysis of median time PFS, 27 months was determined for high Ki-67 compared to 90 months for patients with low Ki-67 signals. Thus, in the light of recent cut-off values used for the Ki-67 staining in different stages and subtypes of OC including our study, we can conclude that Ki-67 values increase continuously with increasing malignancy and do not jump in huge steps which reflects the observations on a similar trend of Ki-67 values in breast cancer (53).

In our protein and mRNA evaluation, both proliferation markers Ki-67 and Plk1 are significant predictors of PFS in early-stage OC. In recent years, PFS has come to the forefront as a primary endpoint, replacing OS in clinical trials in the development of cancer agents, particularly in phase II or III trials, because PFS is a superior clinical aspect compared with OS. Disease progression can be detected earlier than the expiration of candidates, which is associated with reduced encumbrance in fewer candidates and shorter trial durations. Prolonged PFS that postpones tumor-associated morbidity can ameliorate quality of life. The transcriptome-based PFS showed pronounced differences for Ki-67 (low/high: HR 8.91) and Plk1 (low/high: HR 5.07), this trend was also obvious in the analysis of OS for Ki-67 (low/high: HR 3.78) and for Plk1 (low/high: HR 3.6). The prognostic power of the RNA expression data was significantly higher compared to the data derived from the immunohistochemical analysis that cover (low/high: HR 1.45) for Ki-67 and (low/high: HR 1.48) for Plk1 in case of PFS and (low/high: HR 1.01) for Ki-67 and (low/high: HR 1.74) for Plk1 in OS.

We are aware that additional independent cohorts of early-stage OC patients, including endometroid, mucinous, and mixed tumor subtypes, need to be analyzed before a clinical use of cDNA arrays for the determination of Ki-67 and Plk1 levels can be recommended. However, different microarray assays have already been developed as gene expression classifier for the analysis of different cancer types including benign/suspicious thyroid nodules, breast cancer or chronic lymphocytic leukemia and are already in clinical use (54). Thus, the DNA microarray technique has become a versatile tool for expression analysis of clinically relevant markers and might also become helpful for the evaluation of Ki-67 or Plk1 in early-stage OC.

Interestingly, according to the cDNA array data the PFS of OC patients at stages III/IV (Ki-67, low/high: 15.8/17.5 months vs. Plk1, low/high: 16/17.5 months) was moderately better in patients with high expression of both markers. A similar trend was observed for the analysis of OS with Ki-67 (low/high: 40.4/43.9 months) and Plk1 (low/high: 38.7/45.2 months). This observation indicates a qualitative change in the role of proliferation for the development of OC. “Sustaining proliferative signaling” is a hallmark in cancer, but it might play a more dominant role in early-stages of OC than in late stages, where “activation of invasion/metastasis” or “resistance to cell death” take over in the overall clinical picture of OC. In the light of those thoughts, Ki-67 and Plk1 as targets for OC (55–57) come in consideration for early-stage OC in addition to their roles as diagnostic markers. Ki-67 is currently being tested in pre-clinical trials and small molecule inhibitors of Plk1 have received break-through designation by the FDA for clinical trials. Plk1 has already been targeted in clinical trials for OC in comparison to paclitaxel (39). While we demonstrate that high expression of Ki-67 or Plk1 correlates with bad prognosis in stages I/II, the trend for both markers seems to turn around in stages 3/4. As the inhibition of Plk1 alone or in synergistic approaches was very well-suited to induce cell death in OC cells (56), the new data suggest that Ki-67 and Plk1 are attractive targets in particular for the treatment of early-stage OC patients.

In summary, our data on two prominent markers of cellular proliferation, Ki-67 and Plk1, based on the measurements of mRNA and protein expression by using cDNA arrays and immunohisto-chemistry highlight their prognostic relevance in early-stage OC. We suggest that our results derived from Ki-67 and Plk1 can contribute to the stratification of patients in stages I/II which might help to tailor therapeutical strategies. Although or findings are based on a unique cohort of clinically comparable serous patients (*n* = 243), before being implemented in clinical practice, however, our findings require further validation in additional histological subtypes of early stage carcinoma and in serous subtypes of advanced stage patients. The expression of Ki-67 was independent of PLK1 expression. Our findings suggest that Plk1 expression may reflect the degree of malignancy beyond the degree of proliferation in OC. Interestingly, the inhibition of PLK1 and Ki-67 might represent an interesting new targeted approach for the chemotherapy of early-stage OC.

## Data Availability Statement

Publicly available datasets were analyzed in this study. This data can be found here: GEO (https://pubmed.ncbi.nlm.nih.gov/), TCGA (http://cancergenome.nih.gov), and Kaplan-Meier Plotter (www.kmplot.com).

## Ethics Statement

The studies involving human participants were reviewed and approved by Ethics Committee of Sichuan University, Ethics approval no. 20180928032. The patients/participants provided their written informed consent to participate in this study.

## Author Contributions

FR, SZ, BG, and KS: conceptualization and methodology. FR, SZ, BG, MR, MS, RM, DM, SB, and KS: validation and investigation. FR, SZ, BG, and KS: formal analysis and writing–original draft preparation. SZ and BG: resources. FR, SZ, BG, MR, MS, SB, and KS: writing–review and editing. SZ, BG, and KS: funding acquisition. All authors contributed to the article and approved the submitted version.

## Conflict of Interest

The authors declare that the research was conducted in the absence of any commercial or financial relationships that could be construed as a potential conflict of interest.
